# Complex dynamics of a dc glow discharge tube: Experimental modeling and stability diagrams

**DOI:** 10.1038/srep08447

**Published:** 2015-02-13

**Authors:** Eugenio Pugliese, Riccardo Meucci, Stefano Euzzor, Joana G. Freire, Jason A. C. Gallas

**Affiliations:** 1 Istituto Nazionale di Ottica, Consiglio Nazionale delle Ricerche, Largo E. Fermi 6, 50125 Firenze, Italy; 2 Departamento de Física, Universidade Federal da Paraíba, 58051-970 João Pessoa, Brazil; 3 CELC, Departamento de Matemática, Faculdade de Ciências, Universidade de Lisboa 1749-016 Lisboa, Portugal; 4 Instituto de Altos Estudos da Paraíba, Rua Infante Dom Henrique 100-1801, 58039-150 João Pessoa, Brazil; 5 Max-Planck Institute for the Physics of Complex Systems, Nöthnitzer Str. 38, 01187 Dresden, Germany

## Abstract

We report a detailed experimental study of the complex behavior of a dc low-pressure plasma discharge tube of the type commonly used in commercial illuminated signs, in a microfluidic chip recently proposed for visible analog computing, and other practical devices. Our experiments reveal a clear quasiperiodicity route to chaos, the two competing frequencies being the relaxation frequency and the plasma eigenfrequency. Based on an experimental volt-ampere characterization of the discharge, we propose a macroscopic model of the current flowing in the plasma. The model, governed by four autonomous ordinary differential equations, is used to compute stability diagrams for periodic oscillations of arbitrary period in the control parameter space of the discharge. Such diagrams show self-pulsations to emerge remarkably organized into intricate mosaics of stability phases with extended regions of multistability (overlap). Specific mosaics are predicted for the four dynamical variables of the discharge. Their experimental observation is an open challenge.

An outstanding problem is to understand and control the complex chaotic behaviors commonly observed in glow discharge plasma tubes. From an experimental point of view, the first observations of deterministic chaos^1^, mixed-mode oscillations and homoclinic chaos in discharge tubes^2^ date back more than twenty years ago. Currently, much attention has been focused on the generic problem of phase synchronization using these devices. In particular, phase synchronization has been demonstrated under different conditions^3^,^4^,^5^. More recently, the transition to chaos and constructive effects of noise, such as stochastic resonance and coherence resonance, have been reported in plasma tubes^6^,^7^,^8^,^9^. All aforementioned works have in common the feature of using discharge tubes specifically built for research: Geissler's tubes, Plücker's tubes, and so on. Conversely, in this work, we present an application of nonlinear dynamics to study the behavior of a popular device not designed specifically for research purposes. Due to the great impact of plasma as a component in modern image viewing devices, the investigation of plasma tubes is of considerable interest. Additional applications of interest involve microfluidic chips proposed for visible analog computing^10^ and the ability of glow discharges to find the shortest way through a maze^11^.

Electrical discharges have been continuously the subject of innumerous works and much is known about them^12^,^13^,^14^,^15^,^16^. From a theoretical point of view, discharges have been traditionally described taking into account the complex processes involved in the plasma recombination and electric conductivity. Such descriptions require the use of coupled partial differential equations involving spatial and time variables, the transport of momentum and energy of plasma components, the continuity equation, diffusion equation, Poisson equation, etc. This means that a realistic description is generally quite involved. However, a fair description of the discharge can be obtained by bypassing most of the aforementioned complexities and focusing solely on the key feature, namely the discharge capacity of conducting electric current. In other words, one may consider a macroscopic volt-ampere characterization of the discharge, regarding then the whole plasma simply as a nonlinear two-terminal component of an electric circuit. Clearly, such approach removes spatial dependencies and reduces the mathematical description to a nonlinear set of coupled ordinary differential equations, a more easy problem to deal with. The quality of this approach, of course, is proportional to the quality of the volt-ampere characterization of the plasma tube.

In this work we explore the dynamical behavior of the plasma tube in the region of glow discharge. We present a phenomenological model based on the macroscopic electrical features, leading to a two-dimensional nonlinear model. Additional considerations, related to the experimental observation of chaos, induce us to introduce additional degrees of freedom. We then check experimentally and through numerical simulations that our model gives a fair representation of the discharge. Finally, we use the model to predict the distribution of self-pulsations in the control parameter space of the discharge. Before proceeding, we stress the fact that although our model is able to explain a number of experimentally measured features of the discharge, it is just a rough approximation of the complex spatial processes underlying the plasma. Over the years, there have been numerous attempts of modeling plasma discharges as circuits. Such models, however, aim to reproduce the plasma with a much higher accuracy than is needed here. For instance, instead of *ordinary* differential equations, more detailed models normally involve *partial* differential equations. Detailed references about generation of low- and high-energy plasmas can be obtained, e.g., in the several specialized books^12^,^13^,^14^,^15^,^16^.

## Results

**Figure 1:**
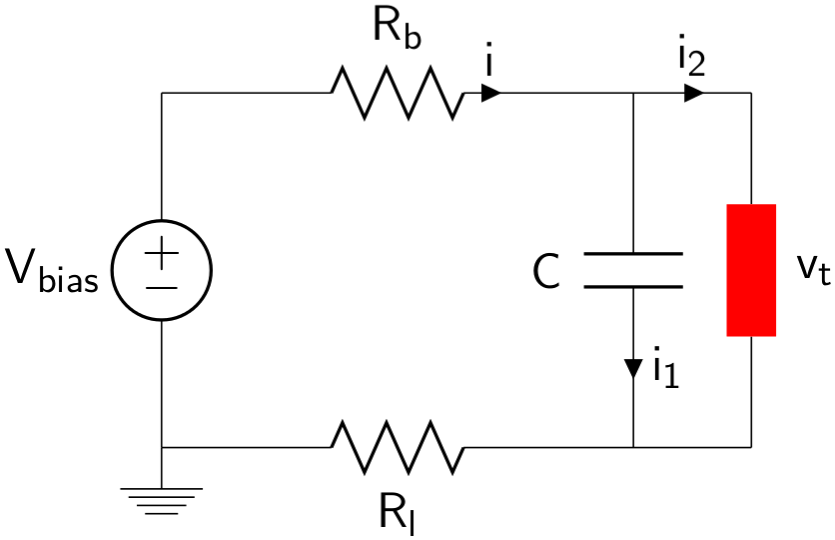
Schematic representation of the experimental setup. The discharge tube is shown as a red element.

The object of our investigations is a plasma tube device of the type commonly used in commercial neon signs and other applications^10^,^11^,^12^,^13^,^14^,^15^,^16^. The tube is filled with neon at a low-pressure of the order of a few *Torr*, and has a length of 50 cm and 2.5 cm of diameter. Both electrodes are identical and can be used as anode or cathode indifferently. The experimental setup is sketched in Fig. 1.

**Figure 2:**
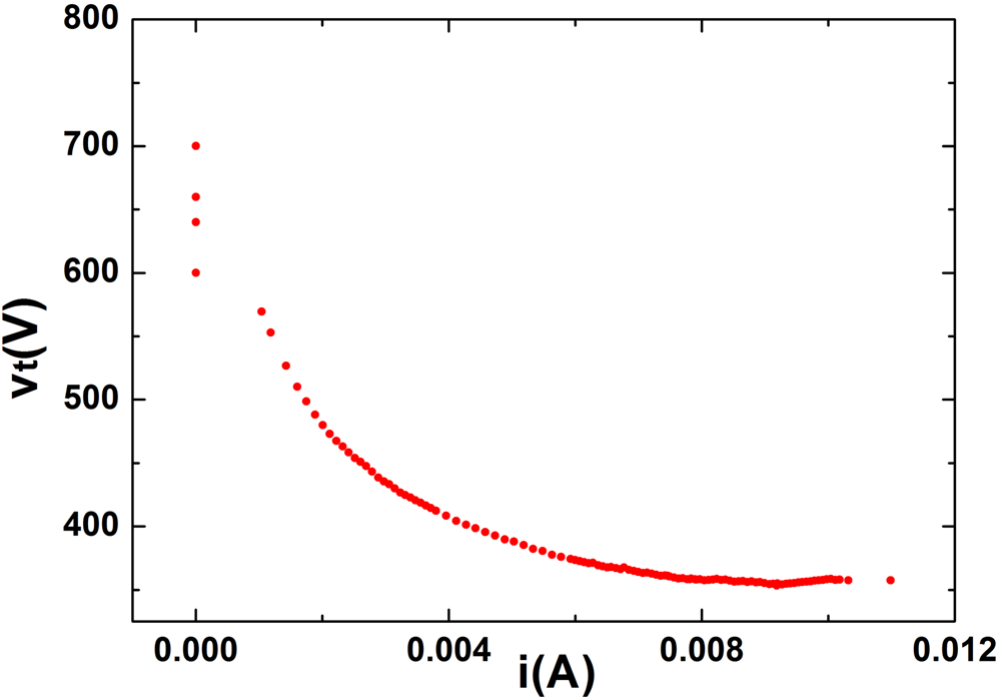
Electrical voltage-current curve of the plasma tube corresponding to the glow discharge region (*i* = *v_l_*/*R_l_*).

An adjustable high-voltage dc source *V_bias_* (*Fluke 415B*) allows us to excite and drive the tube in the glow discharge operating region. The *V_bias_* plays the role of control parameter. Here, *R_b_* and *R_l_* are a 150 kΩ ballast resistor and a 1 kΩ load resistor (nominal values), respectively. The capacitor *C* (2.4 nF), in parallel to the tube, makes the circuit behave as an electrical relaxation oscillator. With this setup, we performed preliminary measurements to determine the electrical nonlinear characteristic of the tube. More explicitly, we measured the voltage drop *v_l_* through *R_l_* (using an Agilent 34401A multimeter) corresponding to several *V_bias_* values. From these data we calculated the discharge voltage across the tube (*v_t_* in our equations) and reconstructed the electrical volt-ampere characteristic curve using the following equation
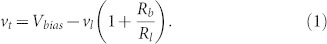
The experimental data are plotted in Fig. 2 where we use the total current *i* instead of *i*_2_, supposing that the current through *R_l_* and the tube are the same. The obtained volt-ampere characteristic curve has a negative slope and is in good agreement with neon lamp curves found in literature^13^.

The range of interest for *V_bias_*, corresponding to the data over the maximum peak plotted in Fig. 2, is 730 V < *V_bias_* < 2000 V. In this operating region we studied the dynamical behavior of the plasma tube. Two distinct signals were experimentally acquired: the discharge voltage across the tube together with the corresponding light emission. A probe ×10 and a photodiode (*New Focus 1621*, not shown in Fig.1) were used. Both voltage signals were recorded with a digital oscilloscope (LeCroy LT342).

**Figure 3:**
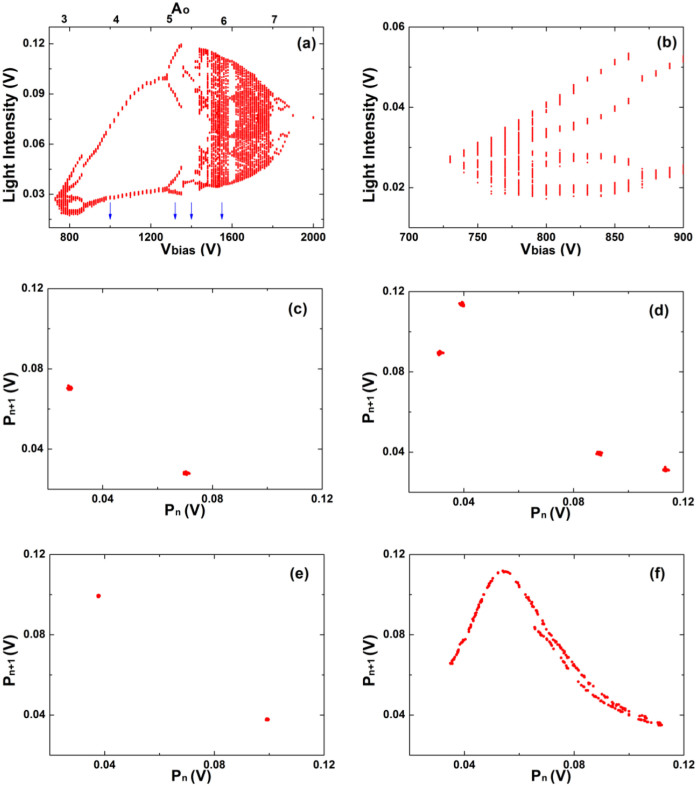
Experimental measurements for *C* = 2.4 nF showing a peak-doubling route to chaos in the discharge. (a) Bifurcation diagram. (b) Enlargement of the leftmost portion of (a). The blue arrows in (a) indicate the values of *V_bias_* where the following return maps were measured: (c) 1000 V; (d) 1320 V; (e) 1400 V; (f) 1550 V.

The recorded temporal sequences were used to construct bifurcation diagrams. Fixing *C* = 2.4 nF, Fig. 3(a) shows a bifurcation diagram where the maxima of the voltage signal are plotted versus the control parameter *V_bias_* and Fig. 3(b) shows a zoom of the range 730 V < *V_bias_* < 900 V. This diagram hints to the presence of a sub-harmonic transition to chaos. Figure 3 (c) shows a return map, namely a plot of successive pairs of maxima of the signal. This return map illustrates a typical period-2 oscillation. Then, by increasing *V_bias_*, one observes a period-4 oscillation and another period-2 oscillation (see Figs. 3(d) and 3(e)), before and after an interior crisis characterized by a sudden decrease in the relative amplitude of the voltage signal (see Fig. 3(a) around 1350 V). Lastly, Fig. 3 (f), shows a structure resembling somewhat the familiar Hénon chaotic attractor.

**Figure 4:**
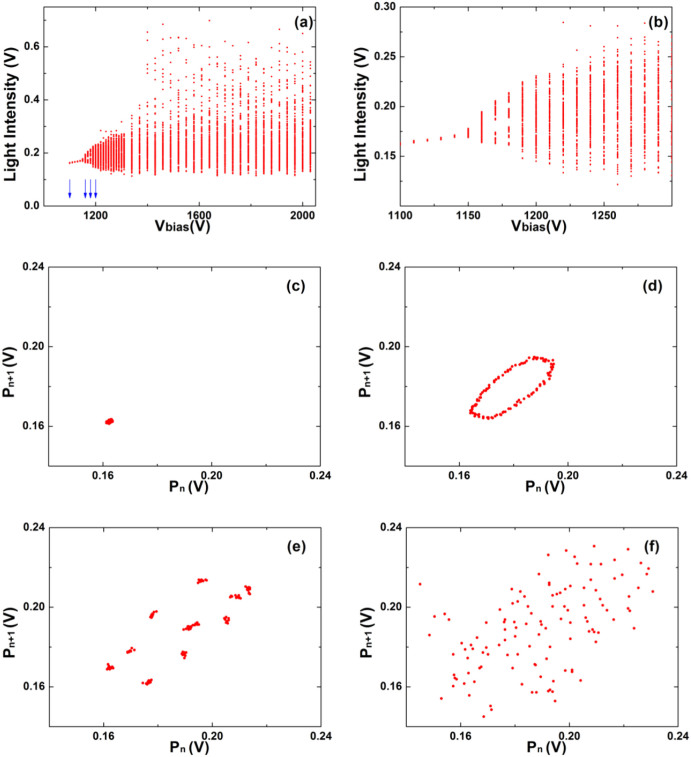
Experimental measurements for *C* = 4.8 nF evidencing a quasiperiodicity route to chaos. (a) Bifurcation diagram. (b) Enlargement of the leftmost portion of (a). The blue arrows in (a) indicate the values of *V_bias_* where the following return maps were measured: (c) 1100 V; (d) 1160 V; (e) 1180 V; (f) 1200 V.

Investigating the *V_bias_* range 730 V–900 V allows us to localize a period-one solution at *V_bias_* = 730 V. Here a limit cycle at a frequency of 670 Hz is observed suggesting that we deal with a stationary regime of the plasma characterized only by the frequency of the relaxation oscillator imposed by the capacitor *C*. In absence of the capacitor *C*, as in Ref. (6), the plasma is in a homogeneous state. In fact, a slightly increase of *V_bias_* leads to period-two solutions and to a narrow chaotic region. As an example, at *V_bias_* = 750 V, the corresponding power spectrum reveals a dominant peak at 890 Hz, its near subharmonic at 450 Hz and a weak remnant peak at 670 Hz. This suggest that a two frequency route to chaos or quasiperiodicy scenario is encountered. The second frequency, competing with the relaxation frequency, is peculiar of the investigated plasma. The nature of transition to chaos was more clearly manifested when decreasing the dissipativity, i.e. when the capacitor value is increased from *C* = 2.4 nF to *C* = 4.8 nF. As can be seen in Fig. 4, in this case the discharge displays an elusive quasi-periodicity route to chaos, a scenario which is corroborated by a careful analysis of a torus breaking phenomenon. The bifurcation diagram in Fig. 4 (a) is markedly different from the one in Fig. 3 (a). The Poincaré sections indicate a transition from a limit cycle (Fig. 4 (c)) to a two dimensional torus (Fig. 4 (d)), as characterized by the closed loop section. A further increase of the control parameter leads to a torus breaking (Fig. 4 (e)) and, successively, to a condition of developed chaos (Fig. 4 (f)).

**Figure 5:**
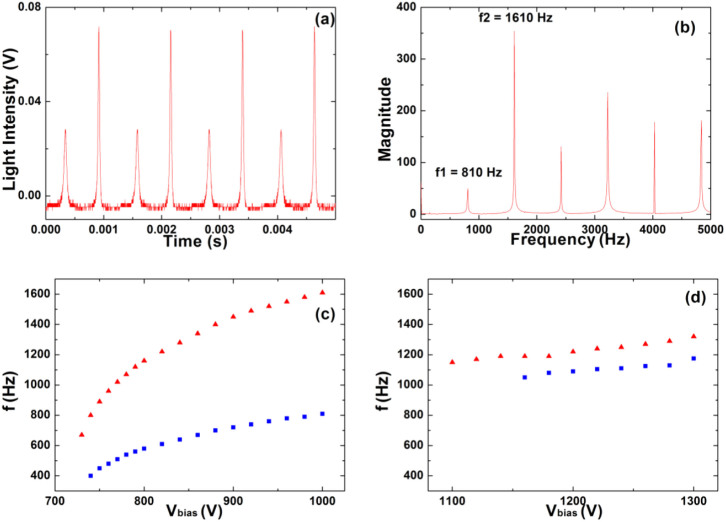
Representative characteristics of the discharge. (a) Temporal sequence, and (b) its magnitude spectrum obtained at *C* = 2.4 nF and *V_bias_* = 1000 V. Relaxation frequency (red) and plasma eigenfrequency (blue) as a function of *V_bias_* for (c) *C* = 2.4 nF, (d) *C* = 4.8 nF.

The temporal behavior and magnitude spectrum associated to the quasiperiodic doubling cascade obtained at *C* = 2.4 nF and *V_bias_* = 1000 V are reported in Fig. 5(a) and 5(b) respectively. Fig. 5(b) clearly displays two peaks, the lower one (plasma eigenfrequency) not being a subharmonic of the relaxation frequency at *f* = 1610 Hz. Fig. 5(c) and 5(d) display the behavior of the relaxation frequency and plasma eigenfrequency as a function of *V_bias_* for *C* = 2.4 nF and *C* = 4.8 nF, respectively. In the first case (Fig. 5 (c)), their ratio (*f_eig_*/*f_mod_*) is near 0.5 while in the second case (Fig. 5(d)) is near 0.89.

We now derive experimentally a macroscopic model of the nonlinear behavior of the plasma discharge. The starting point are Kirchhoff's laws applied to the electrical circuit of Fig. 1. Denoting by *v_t_* and *i*_2_ the discharge voltage and the current through the tube, respectively, it is not difficult to derive the following equations
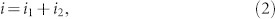

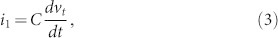

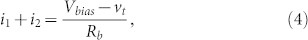
where we disregarded a term containing *R_l_* because 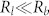. The last two equations yield
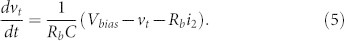
An additional equation governs the voltage drop on the loop
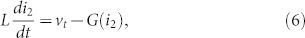
where we introduced the spurious inductance *L* of the tube and its voltage-current characteristic *G*(*i*_2_).

The nonlinear function *G*(*i*_2_) is determined experimentally by measuring voltage drop *v_l_* through *R_l_* (see Fig. 1) and calculating *v_t_* from Eq. (1). In order to obtain a dimension-less form of the previous equations we make the following simplifying assumptions. The experimental electrical characteristics reaches an asymptotic value of about *v* = 360 V on the right border of the normal glow region, we use this value to rescale our equations and to define the first two dimensionless quantities *y* and *g* (hereafter, dropping the subscript, we replace *i*_2_ by *i*)
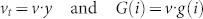
Since the order of magnitude of the current *i* is 10^−3^ A, we introduce a convenient scale factor *α* = 10^−3^ A and a dimensionless variable *x* such that
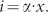
As shown in Fig. 5(a), observing the temporal sequences we can note that the interspikes interval between two successive peaks is almost invariant with respect to our control parameter *V_bias_* and that it assumes the value of about *β*_1_ = 0.5 · 10^−3^ s. We use this value to introduce the dimensionless time variable *t* → *β*_1_ · *t* (maintaining the same symbol *t*). Using two additional definitions, *V_bias_* ≡ *v* · *y_o_* and *β*_2_ ≡ *R_b_C*, from Eq. (5) we obtain
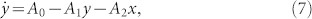
where we used the abbreviations *A*_0_ = *y_o_β*_1_/*β*_2_, *A*_1_ = *β*_1_/*β*_2_, and *A*_2_ = *A*_1_*R_b_α*/*v*.Similar arguments allows us to rewrite Eq. (6) as
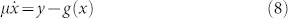
where the parameter *μ* = (*α* · *L*)/(*v* · *β*_1_) is connected to the spurious inductance *L* which cannot be bypassed experimentally.

We assume for *G*(*i*) = *v* · *g*(*i*) a simple mathematical form accounting for the two regions of the characteristic curve, namely
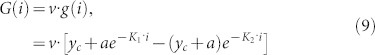
redefining *k*_1_ = *K*_1_ · *α* and *k*_2_ = *K*_2_ · *α* we arrive at the following dimensionless form
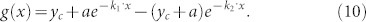
The values of the parameters *y_c_*, *a*, *k*_1_ and *k*_2_ are obtained by fitting the experimental data (Fig. 2). Such fits give *y_c_* = 0.977, *a* = 0.9425, *k*_1_ = 0.4662, *k*_2_ = 26.1, with the quality coefficient of fit being 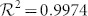, indicating a quite good fit to the data^17^.

Experimentally we observe some windows of chaotic behavior. Since the Poincaré–Bendixson theorem forbids chaos in two-dimensional systems (such the relaxation oscillator described by Eqs. (15) and (16)), to be able to describe the experimental results we need extend the dimensionality of the model by introducing additional degrees of freedom related to the plasma eigenfrequencies. Observing the temporal sequences, we note that in chaotic regime the spikes amplitudes change significantly, in an oscillatory way, but their interspikes intervals are unchanged. This behavior can be reproduced by the following damped oscillator
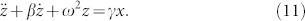
This plasma oscillation is driven by the current and it is rapidly damped when the current decreases to zero. We suggest an oscillatory modulation around the working point to reproduce the experimental time series. In this case, the new electrical characteristic is
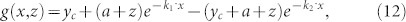
where we fix the parameters *y_c_* = 1, *a* = 1, *k*_1_ = 0.5, and *k*_2_ = 26.

With this, our macroscopic model of the discharge tube is now complete:
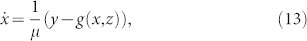

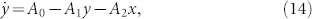

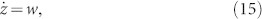

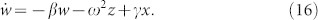
The parameters *A*_0_, *A*_1_, *A*_2_ are related to the experimental configuration adopted. Namely, *A*_0_ is connected to the bias voltage, plays the role of control parameter, and varies in the range [3, 7.8]. Parameters *A*_1_, *A*_2_ depend of the capacitor contained in the electrical circuit, while *μ*, *β*, *ω* and *γ* are free parameters. Concerning the parameter *μ*, we add the following. Since *α* = 10^−3^ A, *v* = 360 V and *β*_1_ = 0.5 · 10^−3^ s, from the definition of *μ* in Eq. 8 we get
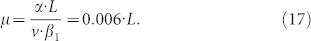
Since it is highly unlikely to have spurious inductances *L* of the order of 1 H, one sees that *μ* is lower than 10^−3^. Note, however, that *μ* can be used to externally control a time-scale of the circuit.

## Discussion

The experimentally derived macroscopic model in Eqs. (13)–(16) can be used to predict numerically the distribution of stable self-generated complex oscillatory patterns in the discharge. Such study serves a few good purposes. On one hand, one may derive a wide-ranging phase diagrams providing a systematic classification of the oscillatory states, of their relative abundance, and of the boundary separating oscillatory phases. Since it is much harder to perform real-life experiments over extended parameter domains than computer simulations, numerically obtained phase diagrams allow experiments to be planed and performed for more promising parameter regions in control space. The availability of numerical predictions provides data against which to compare the reliability of the theoretical description and to improve it where needed.

**Figure 6:**
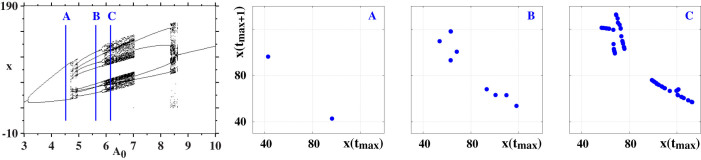
Bifurcation diagram of local maxima of *x* for *γ* = 5 with *ω* varying in the interval *ω* ∈ [6, 7]. Only maxima of x greater than 30 were included. Resolution: 600 × 600. Return maps *x*(*t_max_*) × *x*(*t_max_*_ + 1_) corresponding to the values of *A*_0_ indicated by the blue lines in the bifurcation diagram, namely (*A*_0_, *ω*) = (4.522, 6.217), (5.622, 6.375), (6.161, 6.452).

Using the model in Eqs. (13)–(16) we computed the bifurcation diagram shown in Fig. 6 (a) and three return maps as indicated in the figure. These plots should be compared with the corresponding ones seen in Fig. 3. Note the larger interval of *A*_0_ in Fig. 6 (a). As one sees, while there is a fair overall agreement between Fig. 3 and 6, the bifurcations seen for higher values of *A*_0_ in Fig. 3 (a) display a reverse doubling scenario which is not seen in Fig. 6. This means that the agreement between measurements and modeling deteriorates as *A*_0_ increases. We have also attempted to locate an adequate region of the model to reproduce unambiguously the quasiperiodicity route observed experimentally. While the model can provide signs of quasiperiodicity, clear evidence could not be found.

**Figure 7:**
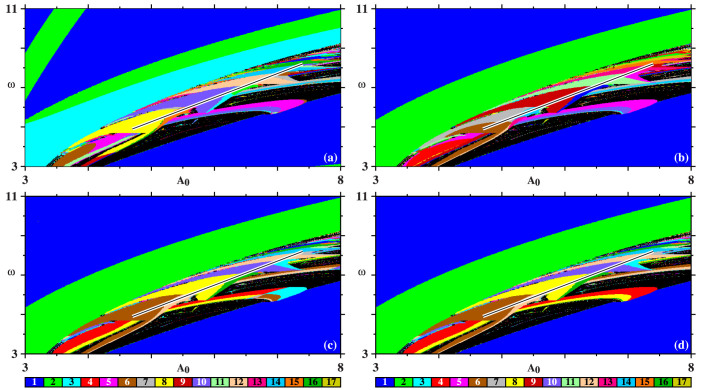
Isospike diagrams obtained by counting the number of peaks in one period of (a) *x*, (b) *y*, (c) *z*, (d) *w*. Integrations were started from the arbitrarily chosen condition, (*x*, *y*, *z*, *w*) = (1, 1, 0.01, 0.01), at *A*_0_ = 3 and continued up to *A*_0_ = 8 by following the attractor.

Our model was also used to compute the *isospike diagrams*^18^,^19^,^20^,^21^,^22^ shown in the four panels of Fig. 7, i.e. phase diagrams depicting for every point in control parameter space the number of spikes contained in one period of the regular oscillations. The colors of the individual panels depict the number of spikes contained in one period of the stable oscillation of each of the four variables *x*, *y*, *z*, *w*, as indicated in the figure caption. Black represents “chaos”, i.e. parameters for which it was not possible to detect any periodicity. Specific details about how these stability diagrams were computed are given below in the *Computational Methods*.

The stability diagrams in Fig. 7 allow one to recognize the rich and intricate interplay between the continuous spike-adding and spike-doubling mechanisms responsible for the complexification of periodic oscillation of the electric discharge. Each variable produces a complex mosaic of colors, showing how the number of spikes self-organize in control space. A significant feature in Fig. 7 is that, although all phase diagrams display the same structure, independently of the variable used to construct them, the individual phases vary in a way that is quite hard to summarize in any means other that by displaying them graphically.

**Figure 8:**
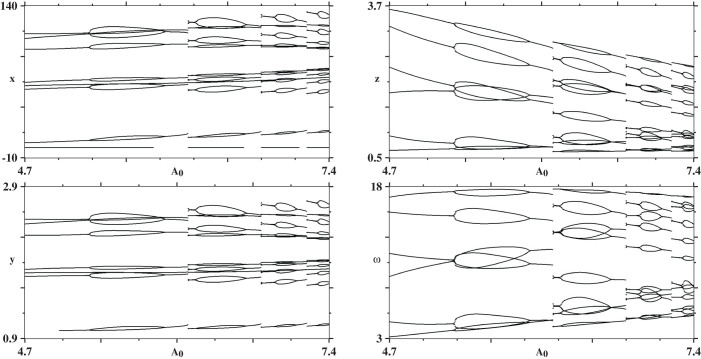
Bifurcation diagrams obtained when varying two parameters simultaneously along the white lines shown in the panels of Fig. 7. Individual panels display 600 × 600 parameter points. Apparently, the discharge is trying to imitate Magrite's *Golconde*, demonstrating the fine line between individuality and group association, and how it is blurred, as in *labyrinth* bifurcations [25].

Figure 8 shows bifurcation diagrams for the four variables of the model, computed by varying two parameters simultaneously along the diagonal straight line segments shown in Fig. 7. While the overall structure of the diagrams is essentially the same, independent of the variable used to count spikes, the number of spikes in the several branches changes considerably, corroborating the sequences recorded in the stability diagram of Fig. 7. Noteworthy are the many jumps observed in the bifurcation diagrams, which signal abundance of multistability in the region, i.e. the possibility of stabilizing distinct attractors according to the initial conditions used. This behavior stresses the richness of the dynamical states supported by the discharge.

In this paper, we reported an experimental study of a low-pressure electrical discharge recording for it the standard doubling scenario as well as a remarkable elusive route to chaos by quasiperiodicity. By characterizing the discharge through a volt-ampere characteristic, we developed a simple model reproducing its basic features. Based on this model, we performed a detailed classification of the oscillatory behaviors, periodic or not, supported by the discharge. By computing stability diagrams for all model variables, we characterized both the size and shape and the unexpected sequential ordering underlying the organization of stability phases. Our diagrams show precisely where the number of spikes changes as a function of the variables used to count them. We found a plethora of stability islands which are simply too complicated to be classified systematically or to be described by other means than purely graphically. Incidentally, we mention that currently there is no method to locate analytically stability phases for nonlinear oscillations of *arbitrary periods* so that the only way to find them is through direct numerical computations. Note that the information in our phase diagrams allows one to effectively *control the dynamics*, namely to select the final dynamical state precisely by performing just a single change of parameters. This is in sharp contrast with conventional methods of controlling the dynamics^23^,^24^, which require pre-investigating unstable orbits, do not include a prescription for the precise selection (targeting) of the final state, and require the permanent application of external perturbations.

In summary, although relatively simple, the macroscopic discharge model reported here can reproduce basic experimental observations and reveals rich and unexpected dynamical facts. Future work should tell if the complex stability mosaics predicted by this model can be also found in experimental diagrams or in predictions derived taking into account spatial phenomena of the discharge as described, e.g. by more sophisticated discharge models based on sets of partial differential equations.

## Methods

**Computational methods**: The isospike diagrams^18^,^19^,^20^,^21^,^22^ in Fig. 7 were obtained by solving the model equations numerically for the following set of parameters: *μ* = 3 × 10^−4^, *β* = 8 × 10^−3^, *γ* = 4.2, *A*_1_ = 1.4, and *A*_2_ = 0.6, where the last two values refer to *C* = 2.4 nF. To this end, we used the standard fourth-order Runge-Kutta algorithm with fixed-step, *h* = 5 × 10^−6^, over a mesh of 400 × 400 equally spaced points. For each value of *ω*, we started the numerical integrations at *A*_0_ = 3 from the arbitrarily chosen initial condition (*x*, *y*, *z*, *w*) = (1, 1, 0.01, 0.01) and then proceed by *following the attractor*, using the last obtained values of the variables to start every new integration involving infinitesimal changes of parameters. The first 400 × 10^6^ time-steps were discarded as transient time needed to reach the final attractor. The subsequent 100 × 10^6^ iterations were then used to compute the number of spikes contained in one period of the oscillations, by recording up to 800 extrema (maxima and minima) of the time series of the variable under consideration, together with the instant when they occur, and recording repetitions of the maxima. As indicated by the colorbar in the figures, a palette containing 17 colors was used to represent “modulo 17” (i.e. recycling colors) the number of peaks (maxima) contained in one period of the oscillations. The computation of stability diagrams is numerically a quite demanding task that we performed with the help of 1536 processors of a SGI Altix cluster having a theoretical peak performance of 16 Tflops. While it is possible to observe period-doubling routes, most of the times what happens is just the addition of a new peak to the waveform (without a corresponding doubling the period). Eventually, after adding several peaks, one reaches a situation where the period roughly doubles a previously observed value.

The bifurcation diagrams in Fig. 8 were obtained by plotting the local maximum values (spikes) of the four variables along the lines shown in the four panels of Fig. 7, namely when tuning *A*_0_ and *ω* simultaneously along the line *ω* = 1.222*A*_0_ – 0.844. In all diagrams, both axis were divided into 600 equally spaced values. As done for the stability diagrams, computations were started at the minimum value of *A*_0_ from the initial condition (*x*, *y*, *z*, *w*) = (1, 1, 0.01, 0.01) and continued by following the attractor using the same integrator and integration step. The first 16 × 10^6^ steps were discarded as transient, while during the subsequent 4 × 10^6^ steps plotting no more than 200 spikes (local maxima) of the variable under consideration.

## Author Contributions

E.P. and R.M. conceived the experiments. E.P. and S.E. performed the experiments. J.G.F. and J.A.C.G. performed the simulations. All authors discussed the results and wrote the manuscript.
